# Beyond hegemonic orders and ‘happy objects’: reimagining women’s empowerment and intersectional leadership in the global south

**DOI:** 10.3389/fsoc.2026.1957241

**Published:** 2026-10-14

**Authors:** Ongeziwe Gift Nodipha, Nthabeleng Innocentia Mdhluli

**Affiliations:** Department of Industrial and Organisational Psychology, University of South Africa, Pretoria, South Africa

**Keywords:** feminist standpoint epistemology, global south, green transitions, hegemonic masculinity, intersectional leadership, women’s empowerment

## Abstract

Women’s empowerment and leadership scholarship has documented women’s exclusion from political, economic, organisational and knowledge institutions in considerable detail. Yet dominant empowerment approaches often treat inclusion as the principal remedy. Women’s advancement is consequently positioned as an individual achievement within structures whose underlying relations of power remain largely unchanged. This conceptual analysis asks: How can structural, affective and epistemic accounts of gendered power be integrated to explain how, and under what conditions, women’s leadership moves from institutional access towards collective and durable institutional transformation in Global South contexts? Through a theory-synthesis design, the article brings into dialogue Connell and Messerschmidt’s multi-scalar account of hegemonic masculinity, Ahmed’s critique of the affective politics of happiness and the ‘happy object’, and Collins’s Black feminist standpoint epistemology, particularly the ‘outsider within’. Intersectionality operates across these traditions as a cross-cutting analytic. The synthesis identifies three generative relational capacities. Structural diagnosis identifies the institutional mechanisms through which inequality is produced and reproduced. Affective refusal contests emotional expectations that protect unequal arrangements. Situated knowledge mobilisation enables socially positioned experience to become collectively interpreted and institutionally actionable knowledge. Their transformative potential is translated through collective capacity into the institutional reconfiguration of authority, resources, recognition, knowledge standards, accountability and work organisation. Six propositions specify the framework’s explanatory pathway. Structural diagnosis redirects action from individual deficits towards institutional mechanisms. Affective sanctions condition whether critique can be expressed and sustained. Situated knowledge gains influence through collective interpretation and institutional connection, while collective capacity enables movement towards institutional reconfiguration. Intersectional distribution determines whether reforms produce structural transformation or selective inclusion. Finally, institutional embedding, durability and recursive feedback determine whether change persists. The South Caucasus is used as a comparative post-socialist boundary illustration to demonstrate the analytical questions the framework generates for gender-just green transitions rather than as a representative Global South case or an empirical test of the framework. The article contributes an integrated, relational and multi-level account of transformative women’s leadership. It explains not merely how women enter institutions, but how leadership may alter the conditions through which institutional power is reproduced.

## 1 Introduction

Women’s empowerment has become influential in gender and development discourse, yet increased representation has not consistently altered the structural conditions through which inequality is reproduced (UN Women, 2020). Empowerment can lose its critical force when struggles over power are translated into programmes centred on individual capacity or measurable inclusion (Batliwala, 2007; Cornwall and Rivas, 2015). This tension persists where women’s advancement is celebrated without sufficient attention to structures governing access to capital, care, legitimacy and opportunity (Lagrasta et al., 2024).

The central problem is therefore not simply whether women gain access to institutions and leadership positions, but whether their participation changes the arrangements determining whose authority is recognised, whose knowledge counts, whose interests shape institutional priorities and whose dissent is tolerated. In South Africa, women attaining senior leadership positions continue to encounter gender stereotypes, work–life pressures and barriers to executive advancement (Mathetha and Dhanpat, 2025), while institutional and resource constraints limit the transformative potential of women’s digital empowerment in the public sector (Shava and Muringa, 2025). Institutional access may therefore expand opportunities without transforming the relations of power governing how those opportunities are exercised.

This distinction is particularly important in Global South contexts, where women’s leadership is shaped by colonial histories, postcolonial state formation, economic restructuring, racialised and classed inequalities, local gender regimes and uneven material resources. The Global South is not treated here as homogeneous (Mohanty, 2003); gendered power and feminist struggles remain historically and politically situated (Rosenkranz, 2024; Wallace, 2023). Universalised models of empowerment may therefore require women to become more productive without redistributing care, more visible without altering institutional power and more confident while leaving intact the conditions that penalise dissent.

This paradox draws attention to what this article terms hegemonic orders: historically and institutionally reproduced arrangements through which particular forms of gendered authority, knowledge and conduct acquire legitimacy while alternatives are subordinated. Connell and Messerschmidt’s (2005) account of hegemonic masculinity provides the principal structural foundation for examining these processes across scales. Hegemonic orders are not assumed to reside only in institutions historically associated with Western modernity, nor is the Global South positioned merely as a setting in which externally theorised structures operate. Gendered authority is also organised through locally and historically constituted formal and informal institutions (Mohanty, 2003; Wiesner-Hanks, 2024). Our standpoint as scholars located within a South African institutional and intellectual context informs this attention to Global South contexts as sites of theory production rather than merely theory application. This situated location is neither neutral nor a guarantee of epistemic authority (Collins, 1986, 1990; Haraway, 1988).

Structural arrangements are sustained not only through formal authority, resources and rules, but also through affective expectations concerning how women should inhabit institutions and exercise authority. Ahmed’s (2010) concept of the ‘happy object’ illuminates how belonging, professional success or recognition may become associated with conformity to culturally approved expectations. Women may therefore gain institutional access while remaining subject to expectations of gratitude, agreeableness, loyalty or composure.

A further challenge concerns whose interpretation of institutional reality becomes authoritative. Collins’s (1986, 1990) Black feminist standpoint epistemology demonstrates how social location shapes what people are positioned to observe and how their knowledge is received. Her concept of the ‘outsider within’ is particularly relevant to women entering institutions from which they have historically been marginalised. Partial inclusion may expose contradictions less visible from dominant institutional standpoints, but such insight does not automatically acquire credibility or influence. Intersectionality further qualifies these processes because relations of race, class, location and other forms of power shape exposure to inequality, the costs of dissent and access to institutional influence (Bilge, 2010; Crenshaw, 1991).

The theoretical problem is not that empowerment scholarship has ignored power or transformation (Batliwala, 2007; Cornwall and Rivas, 2015; Kabeer, 1999). Rather, structural, affective and epistemic dimensions have largely developed through distinct explanatory traditions. Structural theories explain how gendered authority is organised but less fully account for the affective conditions governing whether it can be challenged. Feminist affect theory explains how emotional expectations regulate dissent but not how dissenting experience acquires epistemic authority and institutional leverage. Standpoint epistemology explains how social position can reveal institutional contradictions but not how situated knowledge becomes coordinated collective power. The unresolved problem lies at these explanatory joins.

This article addresses those joins by bringing Connell and Messerschmidt’s (2005) account of hegemonic masculinity, Ahmed’s (2010, 2017) critique of happiness and Collins’s (1986, 1990) Black feminist standpoint epistemology into dialogue. Rather than treating these traditions as interchangeable, the synthesis draws on their distinct explanatory functions to develop three generative relational capacities: structural diagnosis, which identifies institutional mechanisms producing inequality; affective refusal, which contests emotional expectations protecting unequal arrangements; and situated knowledge mobilisation, through which socially positioned experience becomes collectively interpreted and institutionally actionable. These capacities become consequential through collective capacity, which connects them to institutional reconfiguration.

Intersectionality and embedded resource conditions operate across this process. Intersectionality concerns how relations of power are co-produced rather than adding demographic categories to gender analysis (Bilge, 2010; Crenshaw, 1991). Embedded resource conditions concern the material and bodily dimensions of leadership, including care responsibilities, emotional labour, mobility, safety and access to time, finance and organisational infrastructure. Embodiment is therefore treated here as one dimension of embedded resource conditions rather than as a separate cross-cutting analytic. Transformative women’s leadership is consequently not defined by women’s presence or a particular leadership style, but by changes to institutional arrangements governing authority, resources, knowledge, recognition and work. Women’s leadership is not assumed to be inherently transformative; its significance depends on what changes, who benefits and whether altered arrangements become durable.

The framework is developed primarily in relation to Global South contexts. The South Caucasus is subsequently used as a comparative boundary illustration to examine the analytical questions the framework generates when applied to post-socialist green-transition contexts beyond its primary geographical orientation.

### 1.1 Research question

The article is guided by the following research question: How can structural, affective and epistemic accounts of gendered power be integrated to explain how, and under what conditions, women’s leadership moves from institutional access towards collective and durable institutional transformation in Global South contexts?

### 1.2 Conceptual contribution and scope

This article presents a conceptual analysis rather than an empirical study. Its originality lies not in claiming that structural, affective or epistemic analyses of gendered power are individually new. Rather, it lies in specifying how their distinct explanatory functions can be connected and where additional mechanisms are required to explain movement from critical recognition to durable institutional transformation.

The article makes four related contributions. First, it integrates structural, affective, and epistemic explanations of gendered power while preserving their analytical differences. Second, it distinguishes institutional access from transformation and conceptualises women’s leadership as a relational and multi-level process rather than merely a formal position or individual attribute. Third, it develops an explanatory pathway in which structural diagnosis, affective refusal and situated knowledge mobilisation are translated through collective capacity into institutional reconfiguration and, potentially, durable transformation. Fourth, it specifies propositions and boundary conditions that convert this synthesis into a context-sensitive research agenda for empirical examination.

The South Caucasus boundary illustration serves this final purpose. It demonstrates the kinds of analytical questions generated when the framework encounters a post-socialist transitional setting outside its primary Global South orientation. The propositions developed in the article remain theoretically derived claims rather than empirically verified hypotheses.

## 2 Methodological approach to theory synthesis

This article uses a theory-synthesis design to explain how women’s leadership may move from institutional access towards collective and durable institutional transformation. Theory synthesis is appropriate where established perspectives explain different dimensions of a phenomenon but the relationships among those explanations remain insufficiently specified (Jaakkola, 2020). Here, structural theory explains how gendered authority is organised and reproduced; feminist affect theory explains how conformity and dissent are emotionally regulated; and standpoint epistemology explains how social position shapes knowledge and epistemic authority.

The purpose is not to merge these traditions into a unified meta-theory or reconcile their distinct intellectual foundations. Integration occurs at the level of explanatory function: what each tradition explains, where its explanatory reach becomes limited, and what additional relationships are required to connect recognition of inequality with institutional change. Jaakkola’s (2020) theory-synthesis approach provides the overall design, supplemented by Jabareen’s (2009) conceptual-framework analysis and Post et al.’s (2020) guidance on theory development. Principles from integrative reviewing informed literature selection and critical synthesis (Snyder, 2019; Torraco, 2016; van Riel and Snyder, 2024).

The study is therefore a conceptual theory synthesis rather than a systematic review. Its aim is theoretical sufficiency and explanatory development rather than exhaustive bibliographic coverage.

### 2.1 Literature identification and selection

The conceptual corpus was assembled purposively and iteratively. Searches were conducted in Web of Science Core Collection, Scopus and Google Scholar, supplemented by publisher databases, repositories, backward and forward citation tracing, and reference lists of influential publications. Authoritative reports from organisations including the International Labour Organization, UN Women and the World Bank were included where they provided relevant contextual or policy evidence. The literature base was last updated in August 2026.

Searches drew on combinations of terms relating to six domains: women’s empowerment and leadership; hegemonic masculinity and gendered organisations; happiness, affect and dissent; feminist standpoint epistemology and intersectionality; collective agency and institutional change; and women’s leadership in Global South and post-socialist transitional settings. Search terms were combined iteratively as theoretical relationships and tensions emerged during analysis rather than through a fixed systematic-review protocol.

The principal search combinations included terms such as “women empower” OR “women* leadership,” combined with “hegemonic masculinity,” “gendered organisation,” “affect,” “happiness,” “happy object*,” “dissent,” “feminist standpoint,” “outsider within,” “intersectional*,” “collective agency,” “collective action,” “institutional change,” “Global South,” “green transition*” and “post-socialist.” Database syntax was adapted where necessary.

Sources were retained when they defined or refined a central concept, explained a proposed mechanism, introduced a relevant level of analysis, challenged an emerging assumption, offered a rival explanation, or identified conditions affecting whether diagnosis, refusal, knowledge mobilisation or collective action could be exercised and sustained. Foundational texts were retained for their constitutive theoretical contribution, while contemporary literature was used to extend, challenge, or contextualize those arguments. Disconfirming scholarship was deliberately included, particularly evidence showing that women may reproduce unequal arrangements, dissent is not necessarily transformative, marginalization does not automatically generate a critical standpoint, and collective mobilisation may be constrained or captured.

Intersectionality operated as a cross-cutting analytical criterion. Sources were examined for which women were represented, which relations of power were visible, whose knowledge was recognised and whose experiences remained absent. Because the corpus included theoretical, empirical and policy sources, quality was assessed according to conceptual relevance, theoretical clarity, evidential adequacy and contribution to the focal argument rather than through a single methodological scoring instrument.

Theoretical sufficiency served as the closure criterion. Selection continued until additional sources no longer materially altered the principal constructs, proposed relationships, rival explanations, or boundary conditions.

Because the review was designed as a purposive and iterative theory synthesis rather than a systematic review, records were not processed through a fixed screening sequence from which PRISMA-style identification, exclusion and inclusion counts could be reconstructed reliably. The search was instead closed at theoretical sufficiency, when additional searching no longer materially altered the focal concepts, explanatory tensions, capacities or boundary conditions. For this reason, the search information reported in Table 1 documents the scope, sources, selection logic, quality considerations and closure criterion without presenting screening counts that the design was not intended to generate.

**Table 1:** Search and selection transparency.

Element	Approach
Databases	Web of Science Core Collection; Scopus; Google Scholar
Supplementary searching	Publisher databases and repositories; backward and forward citation tracing; reference-list searching
Literature update	August 2026
Language	English-language literature
Main conceptual domains	Empowerment and leadership; gendered authority; affect and dissent; standpoint epistemology and intersectionality; collective agency and institutional change; Global South and post-socialist contexts
Selection logic	Conceptual relevance and capacity to define, connect, challenge, or qualify the framework
Quality consideration	Theoretical clarity, conceptual relevance, evidential adequacy, and contribution to the focal argument
Closure criterion	Theoretical sufficiency

### 2.2 Analytical procedure

The synthesis proceeded through six recursively connected stages.

Stage 1: Institutional access was distinguished from transformation of the arrangements governing authority, resources, recognition, knowledge and work.

Stage 2: Each focal tradition was examined according to its central concepts, object of explanation, conception of power, level of analysis, account of agency and implied process of change.

Stage 3: The theories were compared for complementarity, difference and tension. Hegemonic-masculinity theory explained the structural reproduction of gendered authority but less fully addressed the affective regulation of dissent. Ahmed’s affective analysis explained how institutional attachments and emotional expectations can regulate dissent but did not fully specify how dissenting experience gains collective epistemic authority. Collins’s standpoint epistemology explained how social position shapes knowledge and credibility but did not itself specify how situated knowledge acquires institutional leverage. These differences were retained rather than artificially reconciled.

Stage 4: Three generative relational capacities were derived from the explanatory transitions revealed through comparison. Structural diagnosis captures the identification of institutional mechanisms producing inequality. Affective refusal captures the contestation of emotional expectations that protect unequal arrangements. Situated knowledge mobilisation captures the movement through which socially positioned experience becomes collectively interpreted and institutionally actionable knowledge.

Stage 5: The synthesis identified a further explanatory requirement: capacities must acquire collective leverage before they can alter institutions. Scholarship on collective agency and institutional change therefore informed the specification of collective capacity as the translation mechanism connecting the three capacities to institutional reconfiguration.

Stage 6: Six propositions were derived from the relationships among the generative capacities, collective capacity and institutional outcomes. Five boundary conditions were identified from recurrent qualifications and counter-examples: material resources; political and institutional conditions; intersectional positioning; protection from retaliation; and collective legitimacy and internal accountability. These conditions operate across the proposed pathway by shaping whether the generative capacities can be exercised, translated into collective leverage and sustained through institutional change (Table 2).

**Table 2:** Comparative theory-synthesis matrix.

Theoretical tradition	Core epistemic focus	Primary contribution to interacting capacities
Bosadi theorisation	African-centred womanist critique; contextual and relational agency	Generates relational groundedness and contextual agency; interacts with salutogenesis to frame community-level resilience mechanisms.
Salutogenesis	Sense of Coherence (SOC); origins of health and adaptive coping	Generates comprehensibility and manageability; interacts with critical theory to contextualize resources beyond individualist coping models.
Critical/intersectional theory	Power asymmetries; structural matrix of domination	Generates structural reflexivity; intersects cross-cuttingly with embodiment to expose institutional power dynamics and resource constraints.

The analytical procedure therefore moves beyond comparing three feminist traditions. It makes visible how their distinct explanatory functions generate the three relational capacities, why collective capacity is required as a translation mechanism, and how the six propositions follow from the relationships among these constructs. The five boundary conditions specify where these relationships may be enabled, constrained, or redirected. Section 3 develops the theoretical foundations of these relationships, while Section 4 integrates them into the proposed framework.

## 3 Theoretical foundations

The article draws on three theoretical traditions with distinct but complementary explanatory functions. Connell and Messerschmidt’s (2005) account of hegemonic masculinity explains how gendered authority is organised and legitimized; Ahmed’s critique of happiness explains how affective expectations sustain conformity and make dissent costly; and Collins’s Black feminist standpoint epistemology explains how social location shapes knowledge and epistemic credibility. Intersectionality operates across all three, showing how structural exposure, affective sanction and epistemic authority are differently produced across intersecting relations of power (Bilge, 2010; Crenshaw, 1991). Rather than presenting self-contained theoretical summaries, this section identifies each tradition’s explanatory contribution and limit as the basis for the framework developed in Section 4.

### 3.1 Hegemonic masculinity and the multi-scalar organisation of power

Connell and Messerschmidt’s (2005) reformulation of hegemonic masculinity provides the structural anchor for examining women’s leadership. Hegemonic masculinity is not a fixed personality type or synonym for aggressive male behaviour; it refers to historically specific configurations of gender practice that legitimate unequal gender relations and position particular masculinities as more authoritative than femininities and subordinated masculinities. Gender inequality may therefore persist even where individuals reject overt sexism and organisations formally endorse equality because particular ways of speaking, working and exercising authority continue to appear competent, neutral and legitimate.

Hegemonic masculinity is retained as the structural anchor because its relational and multi-scalar formulation connects institutional arrangements to wider processes through which gendered authority becomes normalised, contested and reproduced. It does not, however, perform all the structural explanatory work. Hearn’s (2004) concept of the “hegemony of men” foregrounds the collective and institutional positioning through which men have historically retained disproportionate control over political, economic and organisational resources. Acker’s (1990, 2006) gendered-organisation and inequality-regime analyses show how these relations become embedded in apparently neutral jobs, hierarchies, evaluation systems and practices of recruitment, remuneration and promotion. Together, these perspectives connect the legitimation of gender hierarchy with the organisational processes through which it becomes routine.

Contemporary research illustrates these dynamics. Women’s attainment of senior leadership positions does not necessarily eliminate stereotyping, work–life pressures or barriers to further advancement (Mathetha and Dhanpat, 2025). Women may also negotiate institutional inclusion through different moral orientations (Aldossari and Murphy, 2024), while organisational actors reproduce and renegotiate gender norms within wider political, religious and cultural conditions (Eger, 2025). Organisations therefore cannot be treated as self-contained arenas of gender inequality.

Gendered power operates across interconnected scales. Household arrangements, community authority and care economies shape the time, mobility and resources available for leadership; organisational structures and informal networks shape opportunity and recognition; state institutions, law and labour markets shape participation; and transnational development, corporate and climate-governance agendas influence valued forms of work and expertise. These scales are mutually constitutive. Organisational expectations of uninterrupted availability, for example, may depend on household arrangements assigning unpaid care disproportionately to women. Connell and Messerschmidt’s (2005) multi-scalar account enables these relationships to be connected without reducing one level to another (Figure 1).

**Figure 1:**
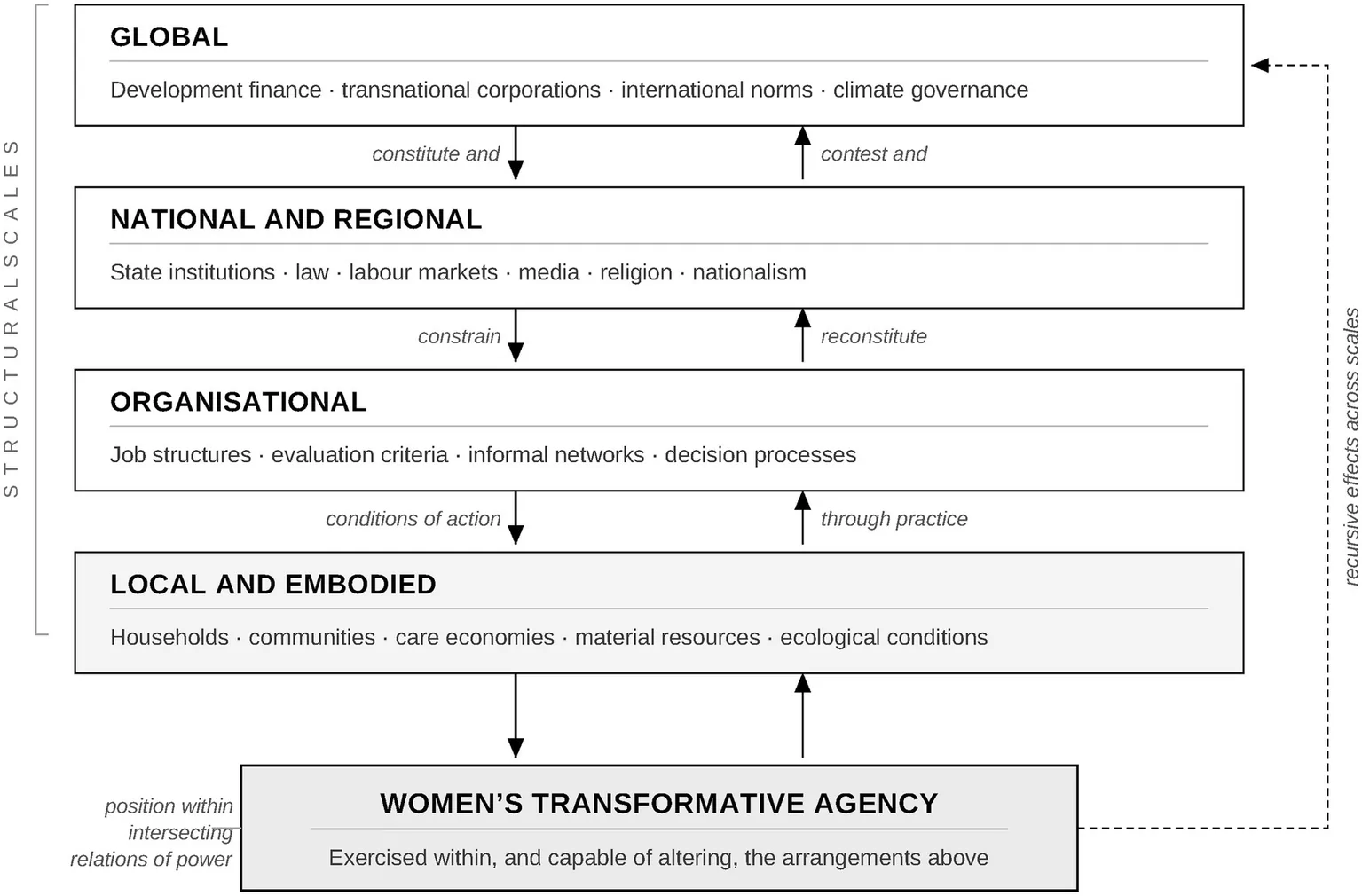
Multi-scalar power and the recursive production of transformative agency. Global, national and regional, organisational, and local and embodied structures are mutually constitutive rather than discrete levels of analysis. Downward arrows denote the constitution of conditions under which leadership is exercised; upward arrows denote contestation and reconstitution through practice. Women’s transformative agency is exercised within these arrangements and may alter them recursively across scales. Intersectional positioning qualifies both exposure to these structures and capacity to act upon them.

A multi-scalar perspective also avoids reducing women’s leadership to a contest between individual women and men. Men occupy unequal positions within hierarchies of masculinity, while women differ by class, race, profession and institutional power and may negotiate or reproduce unequal arrangements where access to authority depends on conformity. Kandiyoti’s (1988) concept of patriarchal bargaining illustrates how women may pursue security or influence within gendered arrangements without dismantling them. Hegemonic masculinity must consequently remain historically and relationally specific rather than becoming a general label for masculine conduct (Hopkins and Giazitzoglu, 2025).

These perspectives explain where and how inequality is institutionally reproduced. Their limit is that structural explanation alone does not account for why recognised inequality may be accommodated, why challenging it becomes emotionally costly or how recognition becomes sustained contestation. This transition provides the basis for structural diagnosis in Section 4 and establishes the need for an affective account of conformity and dissent.

### 3.2 Happiness, compliance and the politics of the ‘happy object’

Ahmed’s (2010) critique of happiness introduces an affective dimension to gendered power. Rather than treating happiness as politically neutral, Ahmed examines how particular identities, relationships and trajectories become associated with socially approved routes to belonging and fulfilment. For women, happiness may become attached to domestic harmony, respectability, organisational belonging, professional gratitude and agreeable femininity. These expectations make some choices appear appropriate while casting dissent as selfish, disloyal or disruptive.

Ahmed’s feminist killjoy names the person who exposes what institutional happiness requires others to ignore. A woman challenging discriminatory procedures, unequal workloads or tokenistic inclusion may consequently be characterised as negative, ungrateful or difficult, shifting attention from the inequality identified to the discomfort produced by naming it. Ahmed’s (2012) analysis of institutional diversity similarly demonstrates how formal commitments to inclusion can preserve institutional comfort: diversity itself may become evidence that an institution is inclusive, rendering continued criticism excessive. Formal permission to participate therefore does not necessarily confer permission to challenge established authority.

Hochschild’s (1983) concept of emotional labour provides an organisational bridge by demonstrating how institutions regulate emotional displays through prescribed feeling rules. Ahmed extends this insight to the emotional attachments that protect institutional arrangements. Women may therefore be required both to perform emotional labour and to absorb the discomfort generated by institutional inequality.

Research on voice and silence further shows that speaking depends on anticipated responsiveness, relational consequences, reputational costs and retaliation rather than confidence alone (Morrison, 2014, 2023). Feminist affect theory adds the question of whose comfort is protected when speaking becomes risky. Women may be heard while remaining reassuring and constructive yet lose credibility when challenging deeper distributions of authority or recognition. Resistance may consequently be indirect and contextually negotiated rather than openly oppositional (Jamjoom and Mills, 2023).

Women’s anger can similarly expose harms concealed by discourses of adjustment and resilience, but may be delegitimized as irrational or incompatible with leadership authority (Murphy, 2024). Dissent should not, however, be romanticized. Anger, complaint and resistance are not inherently progressive, and strategic silence or indirect action may sometimes be safer or more consequential. The analytical concern is therefore the purpose, risks and consequences of dissent rather than conflict itself.

Affective theory thus explains why recognised inequality may not become sustained contestation. Its limit lies further downstream: refusal does not explain how marginalised experience becomes collectively interpreted knowledge, why some claims acquire credibility or how critique gains institutional leverage. This transition provides the basis for affective refusal and establishes the need for an epistemic account of situated knowledge.

### 3.3 Standpoint epistemology and the ‘outsider within’

Collins’s (1986, 1990) Black feminist standpoint epistemology explains relationships among social location, power and knowledge. Social arrangements shape what people are positioned to observe, which questions become salient and whose interpretations are treated as legitimate. This is consistent with the broader feminist insight that knowledge is situated rather than produced from a neutral position outside social relations (Haraway, 1988).

Collins’s “outsider within” emerged from the historically specific positioning of Black women who were included within white households, workplaces and knowledge institutions while remaining socially marginal to them. Such positioning could make dominant assumptions visible because women were close enough to observe institutional arrangements without being fully incorporated into them. Applied cautiously to leadership, the concept helps explain how formal authority may coexist with exclusion from informal networks, dominant standards of professionalism or accepted definitions of expertise.

The concept cannot, however, serve as a universal description of women’s leadership. Women occupy different positions within racial, classed, professional and other relations of power and may themselves reproduce exclusion. Nor does marginalization automatically confer epistemic authority. Social location must be distinguished from an achieved standpoint: situated epistemic advantage becomes critical knowledge through reflection, dialogue and struggle against dominant explanations rather than through group membership alone (Milanovich, 2025; Toole, 2024).

Epistemic-injustice scholarship clarifies what may happen when such knowledge enters institutions. Testimonial injustice occurs when prejudice reduces a speaker’s credibility, while hermeneutical injustice concerns unequal interpretive resources for understanding and communicating experience (Fricker, 2007). Dotson’s (2011) concepts of testimonial quieting and smothering further show how audiences may fail to recognise marginalised actors as credible knowers or induce them to restrict testimony because it is unlikely to be safely understood. Institutions may therefore collect women’s experiences without granting women epistemic authority.

This problem is particularly salient where professional or policy systems privilege externally authorised and technocratic expertise. Knowledge generated through households, informal economies, community organisations and environmental practice may be institutionally marginal despite its practical value. Contextual translation must therefore engage locally meaningful categories rather than assume that classifications developed elsewhere travel unchanged (Wiesner-Hanks, 2024).

The institutional location of the “inside” also requires qualification. Women positioned as outsiders within formal organisations may simultaneously be epistemic insiders within communities, households, informal economies, social movements or local knowledge systems from which formal institutions are partly detached. The institution itself may therefore occupy a partial standpoint where its categories of expertise and legitimate knowledge exclude social worlds on which its decisions depend. Situated knowledge mobilisation is consequently not a process of bringing deficient outsiders into an epistemically complete institution, but an encounter among differently positioned knowledge systems in which institutional authority influences whose account is recognised as legitimate (Collins, 1986, 1990; Fricker, 2007).

Standpoint epistemology thus explains how socially positioned experience may become critical knowledge and why that knowledge can remain institutionally marginal. Its limit is that situated knowledge does not automatically acquire collective force. Influence depends on relationships, resources, protection, shared interpretation and access to consequential decision-making arenas. This transition provides the basis for situated knowledge mobilisation and the need for a mechanism connecting epistemic, affective and diagnostic capacities to institutional leverage.

### 3.4 Intersectionality as a cross-cutting analytic

Intersectionality is not treated as a fourth focal theory or separate stage. It qualifies the structural, affective and epistemic relationships identified above. Crenshaw (1991) demonstrates that relations such as gender and race do not operate as independent layers; their interaction produces positions and inequalities that single-axis analysis cannot capture. Intersectionality therefore shapes structural exposure, affective sanction, epistemic credibility, access to collective resources and the distribution of institutional outcomes (Bilge, 2010; Crenshaw, 1991).

Structurally, race, class, disability, sexuality, nationality, location and employment status shape access to secure work, networks, credentials and authority. Acker’s (2006) inequality-regime approach similarly treats organisational inequalities as interconnected rather than discrete. Nazzal et al. (2024), for example, demonstrate how gender, class and settler-colonial domination combine to produce differentiated constraints and relative privilege among Palestinian women activists.

Intersectionality also shapes the affective and epistemic dimensions of leadership. Expectations of gratitude, respectability and emotional control are racialised, classed and culturally situated, meaning that similar expressions of dissent may be interpreted differently depending on who speaks. Likewise, professional credentials may strengthen some women’s epistemic credibility while knowledge generated through informal work, community organising or care remains marginal. Access to time, finance, mobility, networks and protection also affects who can participate in collective action.

Finally, intersectionality changes how institutional outcomes are evaluated. Reforms ostensibly benefiting women may disproportionately advantage those already possessing education, professional status or class privilege, while women in precarious and informal work remain marginalised. The relevant question is therefore not simply whether women benefit, but which women benefit and whether change redistributes power. This is consistent with Sim and Bierema’s (2025) finding that leadership opportunities are structured through interlocking systems of oppression rather than additive identities.

Intersectionality must nevertheless retain its analysis of power rather than becoming a diversity checklist (Bilge, 2010). Its application must also remain historically and contextually grounded because social categories do not carry identical meanings across societies (Wiesner-Hanks, 2024).

Taken together, the three focal traditions identify a connected explanatory problem. Structural analysis explains how unequal authority becomes institutionalised; affective analysis explains how conformity and dissent are governed; and standpoint epistemology explains how socially positioned knowledge is produced and institutionally received. Intersectionality qualifies each process. Yet structural recognition does not automatically produce contestation, contestation does not automatically confer epistemic authority and situated knowledge does not automatically acquire institutional leverage. These transitions generate the three relational capacities developed in Section 4: structural diagnosis, affective refusal and situated knowledge mobilisation. The capacities do not map mechanically onto individual theories; they emerge from the explanatory relationships and limits revealed through their synthesis.

## 4 An integrated framework of transformative women’s leadership

The synthesis in Sections 2 and 3 identifies three unresolved transitions: structural recognition does not necessarily produce contestation; contestation does not necessarily generate epistemic authority; and situated knowledge does not necessarily acquire institutional leverage. The framework addresses these transitions through three generative relational capacities, a collective translation mechanism, a proximal institutional outcome and a longer-term evaluative condition.

Structural diagnosis, affective refusal and situated knowledge mobilisation constitute the three generative capacities. They are relational rather than individual competencies because their exercise depends on institutional position, social relationships, resources and the responses of other actors. Collective capacity provides the translation mechanism through which these capacities may acquire coordinated institutional leverage. Institutional reconfiguration is the proximal outcome, referring to changes in arrangements governing authority, resources, recognition, knowledge and work. Durable transformation occurs where such changes become enacted, redistributive and sufficiently embedded to persist beyond particular individuals or leadership cycles.

The framework is recursive rather than strictly sequential. Diagnosis may be sharpened through situated knowledge; affective refusal may create space for marginalised knowledge; and collective mobilisation may generate new understandings of institutional power. Figure 2 therefore represents analytical relationships rather than a universal temporal sequence. Intersectionality and embedded resource conditions operate across the pathway, while the boundary conditions specified in Section 4.7 shape whether capacities can be exercised, translated and sustained.

**Figure 2:**
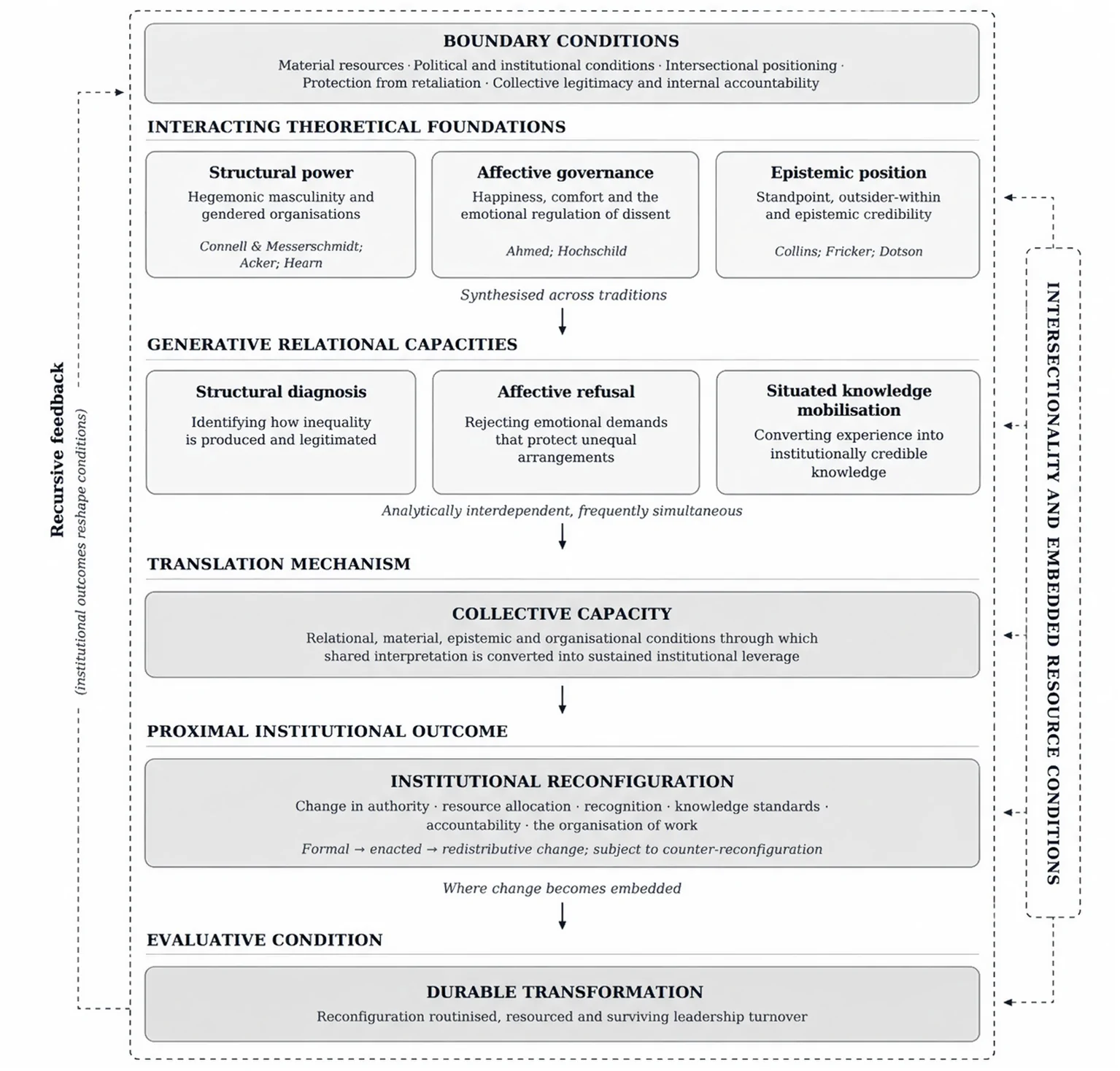
Integrated framework of transformative women’s leadership. Three interacting theoretical foundations jointly inform three generative relational capacities: structural diagnosis, affective refusal and situated knowledge mobilisation. These capacities are analytically interdependent and acquire institutional force through collective capacity as a translation mechanism. Institutional reconfiguration is the proximal outcome; durable transformation is an evaluative condition met where reconfiguration becomes routinised, resourced and able to survive leadership turnover. The dashed return path represents the recursive relationship through which institutional outcomes reshape conditions for subsequent diagnosis, refusal and mobilisation. Intersectionality and embedded resource conditions operate across the pathway, while five boundary conditions shape the conditions under which capacities can be exercised, translated and sustained.

### 4.1 From institutional access to transformation

Institutional access is important to empowerment but is not equivalent to transformation. Access concerns women’s entry into organisations, markets and decision-making spaces; transformation concerns whether participation changes the conditions governing those spaces. Institutions may become more diverse while preserving the rules, networks and resource distributions through which inequality is reproduced.

Three distinctions are important. First, entry differs from influence: formal presence does not necessarily confer influence over agendas, decisions, budgets or accountability. The effects of women managers on workplace inequality vary according to organisational conditions (Fuller and Kim, 2024), while women executives’ influence is shaped by prior institutional experiences and exposure to women with meaningful organisational power (Wiersma S. et al., 2025; Wiersma E. et al., 2025). As technological transitions reshape institutional dynamics, recent scholarship underscores that algorithmic systems cannot be viewed as neutral conduits of efficiency; rather, they require intentional human-centric scaffolding (Lee Y. et al., 2026; Lee S. H. et al., 2026; Wiersma S. et al., 2025; Wiersma E. et al., 2025). In particular, inclusive algorithmic work design demonstrates how diversity-supportive organisational cultures and targeted DEI interventions moderate the psychological demands imposed by AI-driven transformations, thereby mitigating work intensification and safeguarding employee wellbeing (Nodipha, 2026). Second, representation differs from redistribution. Representation changes who occupies institutional positions; redistribution concerns control over authority, resources, time and recognised knowledge. Numerical representation is therefore a possible condition of change rather than evidence of transformation. Third, individual development differs from institutional change. Training, mentoring and professional networking may expand women’s capabilities while leaving underlying institutional arrangements intact. Individualised approaches can displace attention from collective power and systemic inequality (Kark and Buengeler, 2024; Benschop and Lewis, 2025).

Transformative women’s leadership is consequently defined by institutional consequence rather than women’s presence, individual advancement or a particular leadership style. The relevant questions are what changes, whose interests and knowledge shape that change, how authority and resources are redistributed, who benefits and whether altered arrangements endure.

Alternative accounts nevertheless suggest that representation, individual capabilities or strategically positioned actors may produce institutional change under particular conditions. These explanations need not be rejected. Rather, the framework addresses an additional question: whether change redistributes institutional authority, extends across differently positioned women and survives the individuals through whom it was initiated. This distinction becomes particularly important when considering how presence is converted into sustained institutional leverage.

### 4.2 Structural diagnosis

Structural diagnosis is a synthesised relational capacity derived from hegemonic-masculinity theory, gendered-organisation scholarship and feminist institutional analysis. It identifies how inequality is produced and legitimized through relationships, formal rules, informal practices and wider political-economic conditions, shifting attention from what individual women lack to how institutions distribute authority, opportunity, recognition and constraint. Diagnosis therefore moves beyond recognising discrimination to identifying the mechanisms, actors and rules producing unequal outcomes and, consequently, the institutional objects that change must target.

Although diagnosis may begin individually, collective comparison strengthens it. Experiences that appear isolated may reveal recurring relationships among workload allocation, sponsorship, evaluation criteria and leadership recognition when compared across positions and over time. Feminist institutional scholarship demonstrates how formal and informal rules continue to produce gendered effects even where official policies change (Grahn, 2024; Lowndes, 2020).

Diagnosis operates across household, community, organisational, national and global levels and must remain intersectional. The same institutional arrangement may affect women differently according to race, class, disability, nationality, sexuality, location and employment status (Sim and Bierema, 2025). Its immediate consequence is therefore not transformation but a defensible identification of the mechanisms and arrangements requiring change.

### 4.3 Affective refusal

Affective refusal addresses the gap between recognising an unequal arrangement and being able to contest it. Drawing on Ahmed’s (2010, 2017) critique of happiness, Hochschild’s (1983) account of emotional labour and research on voice and silence (Morrison, 2014, 2023), it refers to the capacity to recognise and strategically resist emotional expectations that protect unequal arrangements. These may concern gratitude, harmony, respectability, loyalty, composure or acceptable forms of disagreement.

Affective refusal does not require visible or permanent confrontation. Silence, negotiation, documentation, coalition-building, indirect resistance or selective compliance may be strategic where open dissent carries occupational, relational or physical risk (Jamjoom and Mills, 2023). Its analytical focus is whether actors can challenge the affective terms that make inequality difficult to name and sustain as an object of collective action.

The construct differs from employee voice. Voice concerns communicating concerns or information intended to influence organisational functioning (Morrison, 2014, 2023); affective refusal concerns the emotional conditions imposed on such communication. An organisation may permit speech while requiring criticism to remain sufficiently positive or non-disruptive. Insisting that a complaint be recorded, requesting reasons for an unequal decision or declining to perform gratitude for symbolic inclusion may therefore constitute refusal without requiring withdrawal from the organisation. Refusal is not inherently progressive and must be interpreted against institutional risks, cultural conditions and its consequences for differently positioned actors.

### 4.4 Situated knowledge mobilisation

Situated knowledge mobilisation addresses the transition from socially positioned experience to institutionally consequential knowledge. Building on Collins’s (1986, 1990) standpoint epistemology while retaining the distinction between social location and an achieved critical standpoint, it concerns how experience is collectively interpreted, connected to wider relations of power and translated without removing the interpretive agency of those who produced it. This also reflects the broader feminist insight that knowledge is situated and partial rather than produced from a neutral position (Haraway, 1988).

Mobilisation is therefore more demanding than consultation. Institutions may invite testimony while retaining control over problem definition, evidentiary standards and decisions. Situated knowledge becomes consequential where those who generate it retain meaningful influence over its interpretation and where it enters recognised decision-making and accountability processes (Dotson, 2011; Fricker, 2007).

Five analytically connected processes are involved: articulation, through which previously individualised or silenced experiences are expressed; collective interpretation, through which differently positioned participants identify patterns and differences; critical validation, through dialogue, evidence and competing explanations; epistemic translation, through which knowledge enters institutional processes without losing contextual meaning or interpretive ownership; and institutional uptake, where knowledge influences agendas, policies, resources, work design or accountability.

Mobilisation remains vulnerable to appropriation. Institutions may accept women’s experiences only after professional actors translate them into dominant vocabularies, potentially transferring recognition and benefit away from those who generated the knowledge. Berenstain’s (2016) concept of epistemic exploitation captures this unequal demand for interpretive labour. Mobilisation must therefore protect authorship and interpretive ownership and preserve differences among women rather than manufacturing a unified voice. Whether such knowledge acquires sustained influence ultimately depends on collective capacity.

### 4.5 Collective capacity

The three generative capacities are insufficient for institutional transformation unless they acquire collective force. Collective capacity is therefore the framework’s author-developed translation mechanism rather than a fourth capacity. It refers to the relational, material, epistemic and organisational conditions through which actors interpret problems together, distribute risks, coordinate action and convert diagnosis, refusal and situated knowledge into sustained institutional leverage.

Collective capacity reduces dependence on isolated individuals, provides protection for dissent and connects alternative knowledge and collective demands to consequential decision-making arenas. It is distinct from collective agency, which concerns coordinated action; collective leadership, which concerns distributed influence; and collective capability, which concerns what people can achieve together. Collective capacity instead concerns the conditions that make coordinated and sustained action possible.

This distinction challenges heroic accounts of institutional change. Individualised empowerment may displace collective feminist projects (Benschop and Lewis, 2025), while shared leadership itself operates across unequal terrains of resources and legitimacy (Friedrich et al., 2026). Collective capacity is therefore internally differentiated rather than inherently egalitarian.

Its principal elements are a shared but revisable interpretation of the problem; trust and solidarity; material, informational and temporal resources; coordination across formal and informal networks; representative legitimacy; protection from retaliation; access to consequential decision-making; internal reflexivity and accountability; and capacity to sustain action over time. Internal accountability is essential because women’s collectives may themselves reproduce racial, class, occupational or geopolitical inequalities.

Collective capacity also extends explanations centred on representation or individual critical actors (Childs and Krook, 2009). Increased representation and strategically positioned actors may generate change, but neither establishes whether critique survives the departure, co-option or exhaustion of particular individuals. The relevant question is whether relational and material infrastructure enables claims to be interpreted collectively, risks distributed, less powerful participants represented and institutional pressure sustained. In Global South settings, this infrastructure may extend across workplaces, households, community authorities, informal economies, social movements and development institutions rather than residing within formal organisations alone. Communication infrastructures may support such coordination (Lee Y. et al., 2026; Lee S. H. et al., 2026), but their significance depends on wider distributions of authority and resources.

### 4.6 Institutional reconfiguration

Institutional reconfiguration is the proximal outcome through which collective leverage begins to alter arrangements governing authority, resources, recognition, knowledge and work. Policy adoption, women’s appointment to leadership or the creation of empowerment programmes is therefore insufficient; the question is whether arrangements through which power operates materially change.

Feminist institutionalism demonstrates why this distinction matters. Apparently innovative rules may remain nested within older gendered arrangements (Mackay, 2014), while gender-equality reforms remain sites of institutional contestation (Tildesley et al., 2022). Four related forms of change are distinguished: formal change, where policies, structures or commitments are revised; enacted change, where everyday practices reflect those revisions; redistributive change, where authority, resources and recognised knowledge are reallocated; and durable change, where changes become routinised, resourced and sufficiently embedded to survive leadership turnover.

Institutional change may also undergo counter-reconfiguration, whereby equality initiatives are absorbed, diluted or symbolically displayed while underlying distributions of power remain substantially unchanged. Institutional outcomes must therefore be assessed temporally and distributionally: whether change persists and for whom it redistributes power.

### 4.7 Recursive relationships and boundary conditions

Exercising the three generative capacities is itself materially and affectively costly. Structural diagnosis requires time, information and interpretive labour; affective refusal may entail occupational, reputational and relational risks; and situated knowledge mobilisation requires articulation, translation and coordination. These costs fall unevenly on women already carrying care responsibilities, emotional labour, employment insecurity or economic precarity (Hochschild, 1983; Rai et al., 2014). These material and bodily conditions form part of the embedded resource conditions that shape whether capacities can be exercised and sustained. Transformative agency must therefore be assessed not only by whether capacities exist but also by the resources required to exercise and sustain them.

This prevents the framework from relocating responsibility for transformation onto women. Institutions and collectives must create material, temporal, relational and protective conditions under which diagnosis, refusal and mobilisation can be sustained. Collective capacity can distribute interpretive labour, provide relational and material support and share exposure to retaliation, reducing dependence on the continued sacrifice of particular individuals.

The framework is recursive because institutional outcomes reshape subsequent conditions for agency. Reconfigured reporting systems may strengthen diagnosis; altered emotional norms may reduce the penalties attached to refusal; broader epistemic standards may facilitate situated knowledge mobilisation; and redistributed resources may strengthen collective capacity. Conversely, backlash, institutional capture, resource withdrawal or symbolic reform may weaken these capacities (Mackay, 2014; Tildesley et al., 2022).

Five boundary conditions shape the pathway. Material resources include time, finance, information, mobility, care support and organisational infrastructure. Political and institutional conditions include labour rights, civic freedoms, state capacity and access to decision-making. Intersectional positioning shapes exposure to inequality, sanction, credibility and access to reform (Crenshaw, 1991; Bilge, 2010). Protection from retaliation concerns credible safeguards against occupational, economic, reputational, familial, political and physical consequences. Collective legitimacy and internal accountability concern who may speak for a collective and whether less powerful members influence its priorities. These conditions operate throughout the pathway and specify where, for whom and under what circumstances the proposed relationships may weaken, change form or fail.

### 4.8 Conceptual propositions

The following theoretically derived propositions formalize the principal relationships generated through the synthesis and remain open to empirical examination.

Proposition 1: Structural diagnosis and the target of action.

Leadership practices grounded in multi-scalar and intersectional structural diagnosis are more likely to target institutional mechanisms than practices interpreting inequality as an individual deficit because diagnosis identifies a modifiable object of action. Observable indicators include whether interventions identify specific actors, rules and cross-level relationships; whether targets concern institutional procedures or individual capabilities; and whether the object of change survives the departure of its advocates.

Proposition 2: Affective sanctions and the expression of critique.

Stronger institutional sanctions surrounding gratitude, harmony and emotional conformity constrain whether structural diagnosis and situated knowledge can be expressed and sustained (Ahmed, 2010, 2017; Morrison, 2014, 2023). Affective refusal enables actors to contest or strategically navigate these constraints, while collective protection influences whether refusal can be sustained. The operative mechanism is anticipatory sanction: actors may withhold, soften, delay or redirect critique because of expected consequences. Observable indicators include differences between private and public critique, attribution of problems to complainants rather than procedures, strategic silence or indirect resistance, and whether dissent enters consequential institutional processes.

Proposition 3: Situated knowledge and institutional influence.

Situated knowledge is more likely to influence institutional decisions when collectively interpreted, when those producing it retain interpretive participation and ownership, and when it is connected to recognised decision and accountability processes (Collins, 1986, 1990; Dotson, 2011; Fricker, 2007). The mechanism is control over interpretation: translation by others may reformulate knowledge to fit existing priorities. Observable indicators include whether contributors retain authorship and can contest institutional interpretations, when they enter problem definition, and whether their knowledge alters budgets, technologies or evaluation criteria rather than reports alone.

Proposition 4: Collective capacity as a translation mechanism.

Collective capacity provides the translation mechanism through which structural diagnosis, affective refusal and situated knowledge mobilisation may acquire sustained institutional leverage and contribute to institutional reconfiguration. A central mechanism is risk distribution: dissent concentrated in one individual can be neutralized through removal, promotion or exhaustion, whereas distributed dissent is less easily separated from its institutional object. The relationship should be stronger where collectives possess legitimacy, resources, accountability, protection from retaliation and sustained access to decision processes. Observable indicators include whether claims survive individual advocates, whether less powerful members shape priorities and whether decision-making access persists across leadership cycles.

Proposition 5: Proximity to institutional power and the distribution of reform benefits.

Where access to institutional reform is structured through existing resources, networks, recognised credentials and decision-making proximity, benefits will tend to accrue first to women already positioned closer to institutional power unless explicit redistributive mechanisms alter those conditions. Aggregate improvement may therefore coexist with persistent or widening inequalities. Observable indicators include the distribution of benefits by class, race, disability, occupational status, location and employment security; changes in control over authority and resources rather than participation alone; and whether reforms shift care, emotional labour or other burdens towards women with less institutional power.

Proposition 6: Institutional embedding, durability and recursion.

Institutional reconfiguration is more likely to become durable where formal change is enacted in routine practice, redistributes authority and resources, is supported by accountability and survives resistance, leadership turnover and symbolic absorption (Mackay, 2014; Tildesley et al., 2022). The mechanism is insulation from discretion: changes embedded in budgets, reporting requirements and enforceable procedures are less dependent on individual decision. Observable indicators include survival across leadership transitions, dedicated resources and whether reversal requires formal institutional action. Durable change may recursively expand the legitimacy, resources and protection available for subsequent diagnosis, refusal, knowledge mobilisation and collective action.

## 5 Illustrative application: women’s leadership in green transitions

The South Caucasus provides a useful comparative boundary illustration because Armenia, Azerbaijan and Georgia combine post-socialist institutional legacies, gendered labour-market structures and emerging green-transition agendas. These conditions provide a theoretically informative setting in which to examine how the framework directs attention beyond women’s participation towards the structural, affective, epistemic and collective conditions shaping institutional transformation. The illustration is used to demonstrate the analytical questions generated by the framework rather than to provide an empirical test of its propositions.

Armenia, Azerbaijan and Georgia have distinct political institutions, economic structures, environmental priorities, histories of conflict and gender relations; regional language is therefore used only where comparative evidence is available. Across the region, however, post-socialist institutional restructuring, changing labour markets, persistent gendered divisions of work and emerging green-economy agendas bring state, market, household, community and international-development institutions into interaction. Labour-market gender gaps remain economically significant across the three countries, although their forms differ (Teignier and Cuberes, 2024), while conflict, displacement and uneven institutional development may shape women’s economic security, public participation and exposure to the risks of dissent.

The illustration focuses specifically on gender-just green transitions rather than treating every environmental or economic reform as transformative. A green transition concerns movement towards environmentally sustainable forms of production, energy, employment and resource use. A just transition additionally concerns how the benefits, costs and risks of this movement are distributed, whether affected groups participate meaningfully in decision-making and whether new forms of work are decent and socially protected. A gender-just transition further examines ownership, unpaid care, occupational segregation, knowledge authority, safety and women’s influence over transition priorities. Contemporary scholarship therefore cautions against equating gender justice with the simple addition of women to green jobs or technical programmes (Caretta and Vela-Almeida, 2025; ILO, 2024; Walk et al., 2021).

The analysis is organised around the framework’s principal relationships: how structural diagnosis identifies gendered institutional arrangements; how affective expectations shape the expression of critique; how situated ecological and community knowledge acquires institutional recognition; and whether collective capacity enables these forms of knowledge and contestation to influence institutional arrangements.

### 5.1 Gendered technical and economic authority

Green transitions are frequently framed as technical and economic programmes involving energy, engineering, transport, agriculture, environmental regulation, finance and entrepreneurship. These domains are not institutionally neutral: their professional histories, credentialing systems, investment priorities and leadership structures influence whose knowledge is regarded as technically credible and who gains control over economic opportunities. International guidance on sustainable-energy transitions accordingly identifies women’s recruitment, retention, promotion and decision-making authority as concerns extending beyond numerical participation (ILO, 2024; UN Women, 2023). Structural diagnosis directs attention beyond the number of women entering green sectors, asking instead how technical authority, economic opportunity and institutional legitimacy are organised. Teignier and Cuberes (2024) estimate substantial economic losses associated with gender gaps in labour-force participation and entrepreneurship across the three countries, establishing the regional economic importance of gendered participation without demonstrating that closing participation gaps will automatically redistribute decision-making authority.

Country-specific evidence reveals why participation must be interpreted in relation to occupational structures. In Armenia, women’s relatively high educational attainment has not eliminated field-of-study segregation or care-related constraints: men accounted for 89% of engineering students, fewer than half of working-age women participated in the labour force, and the presence of young children substantially reduced participation (World Bank, 2024a, Executive Summary and Economic Opportunities section). In Azerbaijan, women constituted 10.7% of the energy-sector workforce, 16.5% of workers in transport and storage and 7.8% in construction, and within major state-owned transport enterprises were concentrated in office-based rather than technical or leadership roles; these patterns are linked to occupational stereotypes, care responsibilities and workplace conditions rather than to individual qualifications alone (World Bank, 2024b, Executive Summary and Section 2). Georgia’s Country Gender Equality Profile similarly identifies persistent employment inequalities and includes women’s participation in climate and sustainable-energy transitions among emerging concerns (UN Women Georgia, 2024). These findings do not establish that women are excluded uniformly from environmental decision-making; they show that tarmeniahe institutional conditions from which a green transition begins are already differentiated by employment, care, sector and authority.

Hajiyeva et al. (2024), drawing on nine interviews with women entrepreneurs in Azerbaijan and Georgia, provide context-specific evidence of the challenges women encounter in green entrepreneurship, including limited knowledge of green-economy practices, regulatory complexity, constrained resources and insufficient institutional support. Because the study does not examine Armenia, its findings should not be generalised to the entire region; it is best used to demonstrate how green-enterprise opportunities remain connected to wider financial, regulatory and educational conditions.

These findings generate a different set of analytical questions from those produced by an entry-based model of empowerment. Structural diagnosis would ask who determines green-transition priorities; who controls investment, procurement, land, infrastructure and technology; which qualifications are recognised as technical expertise; how care responsibilities affect access to training and mobility; and whether new green sectors reproduce existing occupational hierarchies. The framework does not assume that women possess an inherent preference for sustainable enterprise, nor that their presence automatically changes transition priorities. What the framework reveals here, and what a participation-based account would miss, is that identical participation figures are compatible with two entirely different institutional situations: one in which women hold decision rights over transition priorities, and one in which they occupy expanded but subordinate positions within priorities set elsewhere. Only the second is visible to an entry-based model, and only the first is transformation.

### 5.2 Affective expectations and public dissent

Green transitions can disrupt established industries, occupational identities and distributions of resources, and women who participate may encounter expectations concerning appropriate femininity, gratitude, respectability and public conduct. Direct regional evidence on the affective regulation of women’s dissent in South Caucasus green-transition institutions remains limited, so this subsection formulates questions for investigation rather than claiming that affective refusal has been observed. Affective refusal therefore functions here as an analytical lens for examining how emotional expectations and anticipated sanctions may shape whether critique can be expressed and sustained.

Available evidence nevertheless identifies social expectations that could shape the emotional conditions of participation. In Armenia, perceptions of gender-appropriate education, employment and public roles continue to influence occupational choice and access to decision-making (World Bank, 2024a). In Azerbaijan, gender stereotypes, family concerns, workplace harassment and masculine occupational cultures are identified as barriers to women’s employment in transport, energy and related technical sectors (World Bank, 2024b), and Hajiyeva et al. (2024) describe comparable constraints affecting women entrepreneurs. These conditions do not prove that women are required to perform gratitude or emotional compliance; they indicate why the question is relevant. Affective analysis would examine whether women are welcomed into green programmes primarily as beneficiaries or symbolic representatives while being discouraged from questioning financing, land use, technical design or the distribution of environmental costs.

The framework therefore asks whether a credible woman leader is expected to remain accommodating and non-confrontational; whether women can challenge technical experts, investors or public officials without losing legitimacy; and whether women’s anger, persistence or refusal is interpreted differently according to class, ethnicity, professional status, location or political connection. Conflict, displacement, employment insecurity and restrictions on civic participation can increase the personal consequences attached to public dissent, so refusal may take the form of strategic silence, documentation, indirect negotiation or coalition-building rather than open opposition. What the framework reveals here is that the apparent absence of contestation cannot be read as consent or satisfaction. A voice-based or participation-based account would record silence as an absence of grievance; the framework treats it as evidence requiring interpretation and asks what the expression of grievance would have cost.

### 5.3 Ecological and community knowledge

Green transitions affect more than technology and employment: they alter access to water, energy, land, transport, food and local livelihoods, and the knowledge required to govern them is distributed across professional institutions, workplaces, households and communities. Feminist political ecology provides a foundation for analysing this distribution. It does not claim that women are naturally closer to nature or uniformly more environmentally responsible; it examines how gendered divisions of labour, access to resources and social position shape environmental experience and knowledge (Rocheleau et al., 1996), and contemporary work on just energy transitions emphasises that women’s environmental knowledge and vulnerability vary by class, location, occupation, care responsibilities and access to political authority (Caretta and Vela-Almeida, 2025; Walk et al., 2021). Women whose labour brings them into sustained contact with agriculture, household energy, water provision, care or community institutions may develop knowledge of particular ecological and social conditions. This is a conditional claim about the relationship between labour, location and experience, not a general claim that all women possess the same environmental expertise.

Situated knowledge mobilisation asks how such experience moves from local awareness into collective and institutional decision-making. Consultation alone is insufficient: women may be asked to describe environmental problems after technical priorities and investment decisions have been set, and their testimony may be collected while state agencies, experts or development organisations retain control over interpretation, authorship and implementation. These risks are acute in development-supported transitions, where the categories defining green entrepreneurship, resilience or technical expertise may be established externally. The framework would therefore ask who defines the problem; who owns the resulting data; whether participants can correct institutional interpretations; whether their knowledge labour is recognised; and whether their contributions influence budgets, technology choices and implementation.

The framework also reverses the conventional deficit assumption. Women may require access to specialised technical, financial and regulatory knowledge, but institutions may simultaneously lack the knowledge produced through women’s work and community experience, so a gender-just transition requires two-directional learning. Mobilisation remains intersectional: urban professional women, rural farmers, informal workers, displaced women and women with disabilities may experience the same transition differently, and the objective is not a single “women’s perspective” but processes through which disagreement can influence priorities. What the framework reveals here is that a capacity-building model diagnoses only one direction of deficit. It asks what women need to learn, and cannot ask what institutions have failed to learn, or why environmental knowledge generated through unpaid and informal labour is systematically classified as anecdote.

### 5.4 Collective capacity and institutional change

Individual entrepreneurship, employment and leadership may improve women’s lives, but the framework does not treat exceptional individual success as evidence of systemic transformation. Movement from participation to institutional change depends on collective capacity, which in green transitions may develop through women’s organisations, cooperatives, professional networks, trade unions, environmental movements, community organisations or cross-sector alliances. Green entrepreneurship is therefore one possible site of transformative leadership rather than its defining form. In terms of the framework, the key question is whether these collective forms convert structural diagnosis, affective refusal and situated knowledge mobilisation into shared institutional leverage.

Regional initiatives show that some infrastructures for collective participation already exist, although programme activity should not be confused with institutional transformation. The Women’s Economic Empowerment in the South Caucasus programme has supported entrepreneurship and agricultural production across the three countries and reported participation in gender-responsive budgeting, private-sector commitments to the Women’s Empowerment Principles and support for policy development (UN Women, 2024). These outputs indicate organised participation and capacity-building, not redistributed authority, enacted policy change or durable transformation.

The framework therefore asks whether women’s organisations possess access to finance, information and decision-making institutions; whether rural, low-income, displaced and disabled women influence collective priorities; whether participants are protected from retaliation; whether organisations can sustain action after donor funding ends; and whether external partners strengthen or displace women’s control over agendas. Collective capacity must be evaluated critically: networks may privilege urban, professional or politically connected women, donor requirements may encourage measurable entrepreneurship outputs while discouraging demands concerning land, public authority, care redistribution or labour rights, and organisations may speak in women’s name without giving less powerful members control over strategy.

Applied to green transitions, institutional reconfiguration could involve women obtaining decision rights in energy and environmental governance; equitable access to green finance, land, procurement and technical occupations; influence over the knowledge standards used in environmental planning; transparent gender-disaggregated accountability; or changes to work and care arrangements that currently prevent sustained participation. Such changes cannot be inferred from the number of women trained, employed or supported to establish businesses. The framework thus extends assessment beyond aggregate economic participation, asking whether women gain influence over transition priorities, whether authority and resources are redistributed, and whether reforms benefit differently situated groups rather than only women already positioned close to institutional power.

Applied across the four subsections, the framework directs attention beyond questions typically foregrounded by entry-based, voice-based and capacity-building accounts: not only whether women participate, but whether they acquire meaningful influence over the terms on which participation occurs. The illustration does not establish the framework’s portability or empirical validity; it shows that the framework produces analytically relevant and locally testable questions in a post-socialist transitional setting. Determining whether the proposed capacities exist, how they are expressed in Armenia, Azerbaijan and Georgia, and whether they produce institutional change requires comparative and locally grounded empirical research.

## 6 Discussion

The discussion returns to the research question by explaining how the integration of structural, affective and epistemic accounts clarifies the conditions under which women’s leadership may move from institutional access towards collective and durable institutional transformation. It also considers how the framework contributes to gender and leadership scholarship, policy and organisational practice, and a future empirical research agenda.

### 6.1 Theoretical contribution

The article’s contribution is not that three feminist traditions can be read alongside one another. It is that each explains a different dimension of a connected process, and that the process becomes analytically incomplete at the transitions between them. Hegemonic masculinity and gendered-organisation theory explain why formal inclusion can leave institutional authority substantially intact, but they do not fully explain why women comply with arrangements they have correctly diagnosed as unequal. Feminist happiness critique explains that compliance by showing how emotional expectations make dissent socially costly, but it does not fully explain why some accounts of inequality acquire institutional credibility while others are received as complaint. Standpoint epistemology explains credibility by locating knowledge within relations of power, but it does not fully explain how situated insight becomes collective leverage. Read separately, each tradition leaves an explanatory transition that the others help to illuminate.

What becomes visible through the integration is the interval between recognising an unequal arrangement and altering it. That interval remains insufficiently specified across the bodies of research brought together in this article, which is why empowerment interventions can be simultaneously well designed and inconsequential. The framework names what has to occur within it: diagnosis must be speakable, speech must be credible, and credibility must be collectively held before it acquires institutional force. Collective capacity is proposed as the mechanism through which this occurs, and its introduction is what allows the framework to explain failure as well as success. Insight without collective protection, voice without authority and policy without embedded practice are not anomalies within this account; they are its predicted outcomes when the translation mechanism is absent.

### 6.2 Contribution to gender and leadership scholarship

The framework differs from four established positions. It departs from individualised empowerment by treating confidence, skills and networks as insufficient where the arrangements governing authority remain unexamined, a concern developed by Kark and Buengeler (2024) and by Benschop and Lewis (2025). It is not a variant of transformational leadership: that tradition concerns a behavioural style and its effects on follower motivation, whereas transformative leadership as used here concerns changes in the institutional distribution of authority, resources, recognition and work. A leader may be transformational in style while leaving every consequential arrangement untouched.

It departs from heroic accounts by declining to place the burden of transforming institutions on individuals who did not design them, and from representation-based models by treating numerical presence as a possible condition of change rather than evidence of it (Fuller and Kim, 2024; Wiersma S. et al., 2025; Wiersma E. et al., 2025). The distinctive claim across all four comparisons is the same: leadership becomes transformative through what it changes institutionally, not through who exercises it or how. This distinction directly advances the article’s second conceptual contribution by clarifying how transformative women’s leadership differs from individualised empowerment, transformational leadership, heroic leadership and representation-based approaches. The framework therefore specifies institutional reconfiguration, rather than leadership style or numerical inclusion, as the criterion against which transformative leadership is assessed.

### 6.3 Policy and Organisational implications

The framework yields an assessment logic that institutions, funders and evaluators can apply. Empowerment initiatives should be assessed against six questions rather than against participation counts: whether women exercise influence over agendas and implementation rather than holding advisory presence; whether decision rights have moved; whether budgets, technology, developmental opportunities and time have been reallocated; whether situated knowledge shapes problem definition rather than being collected after priorities are set; whether dissent is institutionally protected; and whether changes survive leadership turnover and the end of external funding.

Two implications follow. Interventions targeting individual capability should be paired with structural reform, since capability without altered arrangements produces better-equipped participants in unchanged institutions. And reported gains should be disaggregated: reform typically reaches those already closest to institutional power first, so aggregate improvement is compatible with a narrowing distribution of advantage.

### 6.4 Research agenda

The propositions are constructed to be testable, and each implies a design. Propositions 1, 3 suit qualitative construct development and participatory research, since both concern how actors recognise, interpret and retain ownership of knowledge. Proposition 2 requires attention to anticipated rather than observed sanction, which favors longitudinal or critical-incident designs capable of capturing what was not said. Proposition 4 requires process tracing across time because the proposed mechanism concerns how individually or locally generated capacities acquire collective leverage and whether demands survive the departure of particular advocates. Proposition 5 requires disaggregated distributional analysis rather than aggregate measurement. Proposition 6 requires longitudinal designs spanning at least one leadership transition.

Measurement development is a prerequisite throughout. The three generative capacities and the collective-capacity translation mechanism have been specified conceptually but not operationalised, and their indicators will require contextual translation rather than direct transfer. Comparative work across Global South and transitional settings would establish whether the proposed relationships hold or vary by institutional configuration.

### 6.5 Limitations and boundary conditions

Six limitations qualify the framework. It is conceptual: the propositions are theoretically derived and have not been tested. The three traditions do not share a common ontological foundation, and integration at the level of explanatory function manages rather than resolves that difference. Collins’s outsider-within concept was developed through the specific intellectual, racial and labour histories of Black women in the United States, and its use elsewhere requires contextual translation rather than transfer, as Section 3.3 argues. The synthesis was limited to English-language literature, which may have excluded relevant scholarship and locally grounded conceptualisations published in other languages, particularly within the diverse contexts addressed by the framework. The framework’s explanatory claims are also conditional rather than universal. As specified in Section 4.7, material resources; political and institutional conditions; intersectional positioning; protection from retaliation; and collective legitimacy and internal accountability shape whether the proposed capacities can be exercised, translated into collective leverage and sustained through institutional change. These conditions therefore delimit where, for whom and under what institutional circumstances the proposed relationships may strengthen, weaken or take different forms.

The framework also risks essentializing women if the category is treated as internally uniform, which is why intersectionality operates as a cross-cutting analytic rather than a supplementary variable, and why the framework asks which women benefit from any given reform. It risks romanticizing resistance, which Sections 3.2 and 4.3 address directly by insisting that refusal is not inherently progressive and must remain accountable to evidence and consequences. Finally, the illustration is drawn from a single post-socialist region that is not part of the Global South, and it demonstrates only that the framework generates locally testable questions there. Portability across Global South settings therefore remains an empirical question rather than an established property of the framework.

## 7 Conclusion

This article asked how structural, affective and epistemic accounts of gendered power can be integrated to explain how, and under what conditions, women’s leadership moves from institutional access towards durable institutional transformation. The answer proposed here is that the three accounts explain analytically connected rather than competing dimensions of one process, and that the movement between them depends on a mechanism none of them specifies. Structural diagnosis identifies how authority, resources and legitimacy are organised; affective refusal contests the emotional terms that make such diagnosis costly to voice; situated knowledge mobilisation converts experience into institutionally credible knowledge; and collective capacity translates these capacities into leverage capable of reconfiguring institutions. Transformation is durable only where reconfiguration is enacted, redistributive and able to survive leadership turnover.

The framework therefore relocates the question of women’s empowerment. It asks not whether women have entered institutions, but whether the terms on which they participate are theirs to set, and it declines to treat presence, confidence or programme participation as evidence that those terms have changed. Whether the relationships proposed here hold, and how they are expressed across differently configured institutions, is now an empirical question.

The framework’s contribution is therefore both explanatory and conditional. It specifies structural diagnosis, affective refusal and situated knowledge mobilisation as relational capacities whose institutional consequences depend on collective capacity, while recognising that their operation is shaped by material resources, political and institutional conditions, intersectional positioning, protection from retaliation, and collective legitimacy and internal accountability. The South Caucasus illustration demonstrates the kinds of questions this framework can generate in a comparative post-socialist transitional setting, but it neither establishes portability to that region nor provides an empirical test of the proposed relationships. Future research should therefore operationalise the framework’s constructs and propositions across differently configured Global South institutions and comparative transitional settings, examining not only whether women gain access and voice but whether authority, resources, recognition and knowledge are redistributed and whether such changes endure.
